# Influencing Factors of New-Onset Diabetes after a Renal Transplant and Their Effects on Complications and Survival Rate

**DOI:** 10.1371/journal.pone.0099406

**Published:** 2014-06-09

**Authors:** Chaoyang Lv, Minling Chen, Ming Xu, Guiping Xu, Yao Zhang, Shunmei He, Mengjuan Xue, Jian Gao, Mingxiang Yu, Xin Gao, Tongyu Zhu

**Affiliations:** 1 Department of Endocrinology and Metabolism, Zhongshan Hospital, Fudan University, Shanghai, P.R. China; 2 Department of Urology, Zhongshan Hospital, Fudan University, Shanghai Key Laboratory of Organ Transplantation, Shanghai, P.R. China; 3 Evidence Base Medicine Center, Zhongshan Hospital, Fudan University, Shanghai, P.R. China; 4 Department of Endocrinology and Metabolism, People's Hospital Affiliated to Fujian University of Traditional Chinese Medicine (The People's Hospital of Fujian Province), Fuzhou, P.R. China; 5 Department of Cadre's Ward, Fujian Provincial Hospital, Fujian Medical University, Fuzhou, Fujian, P.R. China; Children's Hospital Boston/Harvard Medical School, United States of America

## Abstract

**Objective:**

To discuss the onset of and relevant risk factors for new-onset diabetes after a transplant (NODAT) in patients who survive more than 1 year after undergoing a renal transplant and the influence of these risk factors on complications and long-term survival.

**Method:**

A total of 428 patients who underwent a renal transplant between January 1993 and December 2008 and were not diabetic before surgery were studied. The prevalence rate of and relevant risk factors for postoperative NODAT were analyzed on the basis of fasting plasma glucose (FPG) levels, and differences in postoperative complications and survival rates between patients with and without NODAT were compared.

**Results:**

The patients in this study were followed up for a mean of 5.65 ± 3.68 years. In total, 87 patients (20.3%) developed NODAT. Patients who converted from treatment with CSA to FK506 had increased prevalence rates of NODAT (P <0.05). Multi-factor analysis indicated that preoperative FPG level (odds ratio [OR]  =  1.48), age (OR  =  1.10), body mass index (OR  =  1.05), hepatitis C virus infection (OR  =  2.72), and cadaveric donor kidney (OR  =  1.18) were independent risk factors for NODAT (All P <0.05). Compared with the N-NODAT group, the NODAT group had higher prevalence rates (P < 0.05) of postoperative infection, hypertension, and dyslipidemia; in addition, the survival rate and survival time of the 2 groups did not significantly differ.

**Conclusion:**

Among the patients who survived more than 1 year after a renal transplant, the prevalence rate of NODAT was 20.32%. Preoperative FPG level, age, body mass index, hepatitis C virus infection, and cadaveric donor kidney were independent risk factors for NODAT. Patients who converted from treatment with CSA to FK506 after a renal transplant had aggravated impairments in glycometabolism. Patients with NODAT were also more vulnerable to postoperative complications such as infection, hypertension, and hyperlipidemia.

## Introduction

Since the first renal transplant was performed in the United States in 1954, the number of renal transplant recipients has greatly increased along with the continuous promotion and maturation of the renal transplantation technique. In China, the first renal allotransplant was performed in 1960, and more than 5,000 renal transplants are now conducted in this country each year [1], [2]. The survival time of renal transplant recipients has gradually increased because of the improved survival rate during the perioperative period and enhancements in treatment with anti-rejection drugs [3]–[6]; as a result, long-term complications and the quality of life of transplant recipients have recently received more attention.

New-onset diabetes after transplantation (NODAT) is an important complication after a renal transplant and is officially considered a risk factor for patients undergoing a renal transplant according to the 2003 NODAT international consensus guidelines [7]. Although there is evidence that novel glycometabolism and several chronic diabetic complications are improved by kidney transplantation [8]–[12], NODAT severely affects the quality of life and long-term survival rate of renal transplant recipients [13]–[16]; NODAT is the major factor leading to dysfunction of the renal graft and patient death and is a risk factor for cardiovascular diseases in these patients [17]–[19]. Several in-depth clinical and animal studies have been conducted to study the prevalence rate, risk factors, and pathogenesis of NODAT [20], [21], [34], [35]. With the increasing number of patients with extended survival, the long-term influence of NODAT has been gradually emerging; thus, in recent years, the focus of research has shifted to long-term complications and their influences on continuing human/renal survival.

Our clinical investigation was based on detailed and accurate data from renal transplant cases at Zhongshan Hospital as well as their standardized and orderly postoperative management. Using relevant data from renal allotransplant recipients from January 1993 to December 2008 who survived more than 1 year after surgery, combined with data in the literature on the prevalence rate, outcome, and relevant risk factors for NODAT, this study evaluated the influence of NODAT on complications and long-term survival of these patients and provides a new clinical basis for the prevention and treatment of NODAT.

## Subjects and Methods

### 1. Subjects

We retrospectively analyzed the records of 709 patients who underwent a renal transplant at Zhongshan Hospital, Fudan University, from January 1993 to December 2008. The following patients were excluded: 162 patients with unclear data on preoperative medical history and missing postoperative follow-up information, 75 patients whose renal graft survived less than 1 year after surgery, 10 patients with a combined liver-kidney transplant or other multi-organ transplant, 16 patients who underwent 2 or more renal transplants, and 18 patients who were diabetic before surgery. The remaining 428 non-diabetic patients who underwent a renal transplant for the first time and had a renal graft survival time of more than 1 year and complete data were included for analysis. This study was approved by the institutional review board of Zhongshan Hospital, Fudan University, and all participants provided written informed consent.

### 2. Methods

#### (1) Collection of data

Hospitalization and outpatient data from patients who underwent a renal transplant at Zhongshan Hospital, Fudan University, between January 1993 and December 2008 were collected. Basic preoperative data included general data (gender, age, BMI), preoperative examination, history of smoking and family history of diabetes, primary renal diseases and source of donor kidney, and preoperative biochemical indicators. Data on the patient's condition during the perioperative period included intraoperative immune induction, initial immunosuppressant regimen, acute rejection (AR), and recovery of renal function. Postoperative follow-up data included postoperative fasting plasma glucose (FPG) level, blood lipid level, glycated hemoglobin level, renal function, hepatitis B virus/hepatitis C virus/cytomegalovirus markers, postoperative immunosuppressant maintenance regimen, drug dosage and concentration, complications (AR, history of major infections, tumor), renal graft (normal function, dysfunction), and patient survival or death. Endpoints were defined as death of patients or dysfunction of renal grafts.

#### (2) Immunosuppressant treatment regimen after a renal transplant

A triple regimen of cyclosporin (CSA) plus mycophenolate mofetil/azathioprine plus glucocorticoids or tacrolimus (FK506) plus mycophenolate mofetil/azathioprine plus glucocorticoids was used for postoperative immunosuppressant treatment. Primary CSA-treated patients were converted to treatment with FK506 or rapamycin if adverse reactions such as liver and kidney poisoning, insensitivity to CSA, significant gingival hyperplasia, chronic rejection, or malignant tumors were observed. Drug concentrations were monitored during follow-up, and drug dosages were adjusted on the basis of the plasma drug concentrations and the particular conditions of the patients. Peak concentration was monitored for CSA and trough concentration was monitored for FK506; plasma drug concentrations were maintained within the therapeutic window (Table 1).

**Table 1:** Reference plasma drug concentration (ng/ml) of immunosuppressor in different periods after transplantation.

Drug	1 month after transplantation	2–3 months after transplantation	4–6 months after transplantation	7–12 months after transplantation	1 year after transplantation
Cmin of FK506	6–15	8–15	7–12	5–10	7–9
Cmax of CSA	1200–1500	1000–1200	800–1100	700–1000	650–900

#### (3) Blood sugar management regimen

All patients underwent periodic follow-up after surgery as required by the follow-up system at our medical center. FPG and blood lipid levels were measured, and the immunosuppressant regimen was adjusted according to the particular conditions of the patients during follow-up. Patients who were diabetic before a transplant and patients with NODAT were treated and monitored according to the 2005 guidelines for treatment and management of NODAT [22].

#### (4) Criteria of relevant indexes

Patients were considered to have NODAT if they were not diabetic before surgery and did not have an acute glycometabolism disorder after surgery but met the diagnostic criteria for diabetes with a sustained high hyperglycemic state or normal blood sugar level and were currently being treated with insulin or an oral anti-diabetic drug. The diagnosis of NODAT was based on the following diagnostic criteria for diabetes proposed by the American Diabetes Association in 2007: typical symptoms of diabetes with a random blood glucose level ≥11.1 mmol/L, an FPG level ≥7.0 mmol/L, and a 2-hour blood glucose level after glucose load (75 g anhydrous glucose) ≥11.1 mmol/L on an oral glucose tolerance test (OGTT); an FPG level between 5.6 and 6.9 mmol/L is considered impaired fasting glucose (IFG), and a 2-hour blood glucose level between 7.8 and 11.0 mmol/L is considered impaired glucose tolerance (IGT) [23].

Patients may have significantly different recovery of early renal function after a renal transplant. For the purposes of our research, all patients in this study were divided into 4 categories: (1) immediate recovery of renal function: serum creatinine level decreased to less than 140 µmol/L within 7 days after surgery; (2) slow recovery of renal function: serum creatinine level decreased to less than 140 µmol/L after 1 week, but hemodialysis was not needed; (3) delayed recovery of renal function: serum creatinine level decreased to less than 140 µmol/L after 1 week, but postoperative oliguria or anuria was observed and dialysis transition was needed; and (4) impaired recovery of renal function: serum creatinine level was greater than 140 µmol/L 30 days after surgery. Delayed recovery of renal function was not included in the cases of impaired recovery of renal function.

On the basis of the time of NODAT, patients were considered to have early NODAT (new-onset diabetes within 1 year after a renal transplant), late NODAT (new-onset diabetes more than 1 year after a renal transplant), or no NODAT (N-NODAT) (no NODAT was observed after surgery and until the endpoint of follow-up). According to whether NODAT existed continuously, the patients were considered to have transient NODAT (T-NODAT) (NODAT persisted for at least 3 months and returned to normal during follow-up) or persistent NODAT (P-NODAT) (NODAT existed during follow-up and to the endpoint).

#### (5) Statistical analysis

SPSS 16.0 and SAS 8.2 software packages were used for statistical analysis. Measurement data are expressed as mean ± standard deviation, and count data are expressed as values and percentages. Intergroup comparisons of measurement data that are in accordance with normal distribution and show homogeneity of variance were performed using the *t* test or single-factor analysis of variance; otherwise, the grouped Wilcoxon test was used. Count data were analyzed using chi-square test or Fisher exact probability test. P < 0.05 was considered to be a statistically significant difference. Analysis of the influences of immunosuppressant therapy on FPG and blood lipid levels before and after immunosuppressant conversion was performed using *t* test. Logistic multivariate stepwise regression analysis was used for multi-factor analysis of risk factors for NODAT. The Kaplan–Meier method was used for survival analysis to estimate the survival rate, and the stratified log-rank test was used to compare the 2 survival curves. The Cox proportional hazards model was used to analyze factors that affect the survival of renal transplant recipients.

## Results

### 1. General preoperative conditions of patients with and without NODAT

After eliminating 281 of the 709 renal transplant recipients on the basis of the exclusion criteria, 428 non-diabetic patients who underwent a renal transplant for the first time and had a renal graft survival time of more than 1 year and complete data were included for analysis. Patients were divided into the NODAT and N-NODAT groups according to their postoperative FPG levels; the general preoperative condition of the patients is shown in Table 2. Compared with the N-NODAT group, the NODAT group was older, had higher body mass index (BMI) values, and had higher proportions of patients with a preoperative history of smoking and a family history of diabetes in first-degree relatives. HCV and CMV infection rates were higher in the NODAT group than in the N-NODAT group, whereas there was no significant difference in hepatitis B virus infection rates between the 2 groups. Preoperative total cholesterol (TC) and triglyceride (TG) levels were both higher in the NODAT group than in the N-NODAT group.

**Table 2:** Characteristics of patients with NODAT versus N-NODAT before transplantation.

Clinical index	NODAT (n = 87)	N-NODAT (n = 341)	P-Value
Gender (Male)	60(69.0%)	228(66.9%)	0.798
Age (year)	45.61±9.86	38.89±12.67	0.004
BMI (kg/m^2^)	24.84±4.12	20.97±2.81	0.010
History of smoking	25(28.7%)	37(10.9%)	<0.001
History of diabetes	20(23.0%)	20(5.9%)	<0.001
**Type of dialysis**			
Hematodialysis	68(78.2%)	277(81.3%)	0.505
Peritoneal dialysis	5(5.7%)	30(8.8%)	
Without dialysis	14(16.1%)	34(10%)	
Duration of dialysis	423.67±536.42	344.65±439.45	0.154
HBV infection	11(12.6%)	28(8.2%)	0.212
HCV infection	18(20.7%)	27(7.9%)	0.001
CMV infection	15(17.2%)	27(7.9%)	0.020
FPG (mmol/L)	5.48±0.79	4.39±0.55	0.002
TC (mmol/L)	4.58±1.26	4.05±0.96	0.045
TG (mmol/L)	1.86±1.06	1.43±0.76	0.024

Abbreviations: NODAT: new onset diabetes after transplantation; N-NODAT: no NODAT; BMI: body mass index; HBV: hepatitis B virus; HCV: hepatitis C virus; CMV: cytomegalovirus; FPG: fasting plasma glucose; TC: cholesterol; TG: triglyceride.

### 2. Prevalence and outcome of NODAT

**Figure 1:**
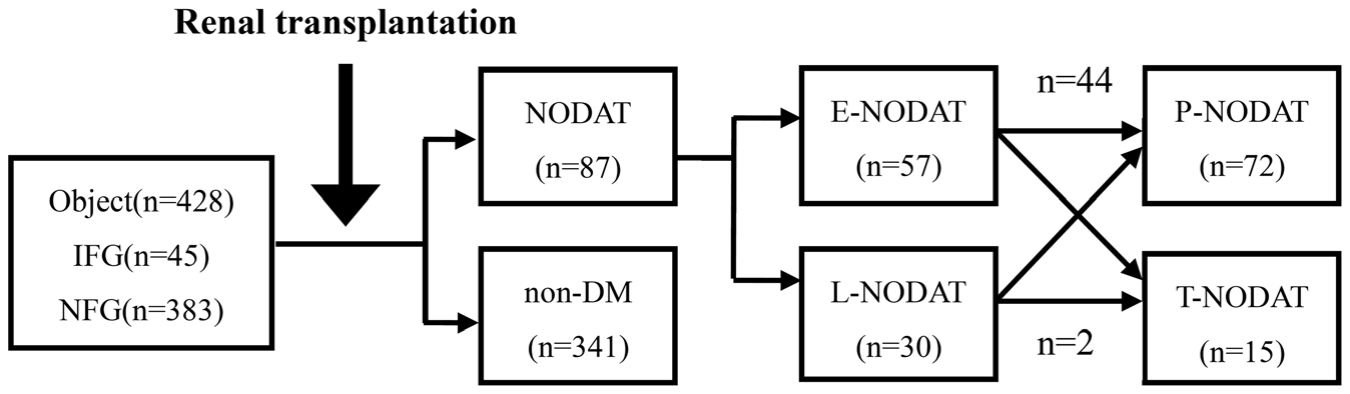
Prevalence and outcome of NODAT. Abbreviations: IFG: impaired fasting glucose; NFG: normal fasting glucose; NODAT: new onset diabetes after transplantation; E-NODAT: early-NODAT; L-NODAT: late NODAT; N-NODAT: no NODAT; T-NODAT: transient-NODAT; P-NODAT: persistent-NODAT.

The 428 patients who were non-diabetic before surgery (including 45 patients with IFG and 383 patients with normal fasting glucose levels) had an average follow-up time of 5.65 ± 3.68 years until the endpoints of follow-up. Eighty-seven (20.32%) of these patients had onset of NODAT, and 57 of these cases occurred within 1 year (accounting for 65.5% of all cases of NODAT). Among all patients with NODAT, 72 had persistent NODAT and 15 (17.2%) shifted to normal fasting glucose or IFG (Figure 1).

### 3. Risk factors for NODAT

#### (1) Risk factors for NODAT

Risk factors that might be involved in postoperative onset of NODAT were analyzed using single factor analysis, and the results are shown in Table 3. The proportion of patients with a cadaveric donor kidney was higher in the NODAT group than in the N-NODAT group. The early postoperative FPG level 7 days after surgery was higher in the NODAT group than in the N-NODAT group. The proportion of patients treated with basic CSA or FK506 immunosuppressant regimens at discharge was not statistically significant between the 2 groups. However, during the course of disease, 19 patients (21.8%) in the NODAT group and 30 patients (8.8%) in the N-NODAT group converted from treatment with CSA to FK506, which was significantly different between the 2 groups. In the NODAT group, some of the patients experienced onset of NODAT after adjustment of their immunosuppressant regimen (conversion from CSA to FK506). In terms of the comparison of anti-rejection drug dosage and plasma drug concentration, the daily dosage of corticosteroid maintenance therapy was not significantly different between the 2 groups. CSA peak concentrations in the NODAT group at 6 months and 1 year were greater than those of the N-NODAT group, and the difference was statistically significant; however, differences in FK506 trough concentrations at each period were not statistically significant.

**Table 3:** Comparison of risk factors between NODAT and N-NODAT (single factor analysis).

Risk factors	NODAT (n = 87)	N-NODAT (n = 341)	P-value
Graft type (cadaveric)	79(90.8%)	249(73.0%)	<0.001
**Recovery of graft function**			
Immediately	52(59.8%)	232(68.0%)	0.258
Slowly	23(26.4%)	62(18.2%)	
Delayed	8(9.2%)	24(7.0%)	
Difficult to recover	4(4.6%)	23(6.7%)	
Induction therapy (antiCD25)	38(43.7%)	171(50.1%)	0.337
Acute rejection	14(16.1%)	45(13.2%)	0.488
FPG 1week after transplant (mmol/L)	6.46±4.24	5.12±1.14	0.005
FK506/CSA on discharge	11/76	31/310	0.317
AZA/MMF	13/74	44/297	0.156
CSA convert to FK506	19(21.8%)	30(8.8%)	0.020
CSA convert to rapamycin	6(6.9%)	25(7.3%)	1.000
**Daily dose of glucocorticoid (mg)**			
On discharge	26.26±4.77	26.73±5.00	0.435
3 months after transplantation	17.42±4.75	16.13±3.26	0.132
6 months after transplantation	13.51±2.70	13.91±3.45	0.573
1 year after transplantation	12.74±3.78	11.47±3.26	0.061
**Cmax of CSA (ng/ml)**			
On discharge	1284.80±282.44	1276.80±367.74	0.853
3 months after transplantation	1016.50±251.64	1002.30±328.35	0.861
6 months after transplantation	1030.80±332.04	906.38±304.97	0.012
1 year after transplantation	929.81±278.86	785.51±226.02	0.006
**Cmin of FK506 (ng/ml)**			
On discharge	11.03±3.39	9.04±3.93	0.144
3 months after transplantation	10.60±7.26	7.79±2.57	0.054
6 months after transplantation	7.85±2.48	7.52±2.01	0.808
1 year after transplantation	7.44±2.28	7.20±2.76	0.836

Abbreviations: NODAT: new onset diabetes after transplantation; N-NODAT: no NODAT; FPG: fasting plasma glucose; AZA: azathioprine; MMF: mycophenolate mofetil; FK506: tacrolimus; CSA: cyclosporin.

##### Risk factors for NODAT (multi-factor analysis)

Logistic multivariate regression analysis was performed to analyze age, BMI, history of smoking, family history of diabetes, source of donor kidney, HCV infection and CMV infection, preoperative TC and TG levels, postoperative immunosuppressant regimen, daily dosage of corticosteroid maintenance for different times of year, and plasma concentration of CSA and FK506. As shown in Table 4, age, BMI, HCV infection, preoperative FPG level, and a cadaveric donor kidney can increase the risk of NODAT.

**Table 4:** Logistic regression analysis of risk factors for NODAT (multiple-factor analysis).

Risk factors	OR	95% confidence interval	P-value
Age	1.10	1.02–1.23	0.044
BMI	1.05	1.04–1.14	0.029
HCV infection	2.72	1.20–6.34	0.008
Cadaveric graft	1.58	1.43–1.90	0.035
Preoperative FPG level	1.48	1.02–1.57	0.036

Abbreviations: NODAT: new onset diabetes after transplantation; BMI: body mass index; HCV: hepatitis C virus.

#### (2) Comparison of risk factors between P-NODAT and T-NODAT

As shown in Table 5, compared with T-NODAT, the P-NODAT group was older, had higher BMI values, had greater proportions of patients with a family history of diabetes and a cadaveric donor kidney, and had higher preoperative and 1-year postoperative FPG, TC, and TG levels; these differences were statistically significant, whereas the concentrations of immunosuppressants showed no significant differences between the 2 groups. It is noteworthy that the prevalence rate of AR in the T-NODAT group was higher than that in the P-NODAT group (P  =  0.043).

**Table 5:** Comparison of risk factors between P-NODAT and T-NODAT.

Risk factors	P-NODAT (n = 72)	T-NODAT (n = 15)	P-value
Age (year)	42.58±9.21	38.73±12.91	0.046
BMI (kg/m^2^)	25.98±3.32	22.17±6.92	0.036
Gender (male)	51(21%)	9(60%)	0.540
History of diabetes	19(26.4%)	1(6.7%)	0.045
History of smoking	23(31.9%)	2(13.3%)	0.213
HBV infection	10(13.9%)	1(6.7%)	0.681
HCV infection CMV infection	16(22.2%)	2(13.3%)	0.526
CMV infection	5(6.9%)	0	0.582
FK506/CSA on discharge	9/63	2/13	1.000
AZA/MMF	11/61	2/13	1.000
CSA convert to FK506	17(23.6%)	2(13.3%)	0.506
CSA convert to rapamycin	4(5.6%)	2(13.3%)	0.275
**Cmax of CSA (ng/ml)**			
3 months after transplantation	1231.90±262.03	1272.10±243.19	0.875
1 year after transplantation	962.19±289.08	816.47±224.28	0.042
**Cmin of FK506 (ng/ml)**			
3 months after transplantation	8.56±2.52	8.90±2.30	0.419
1 year after transplantation	8.68±1.29	7.00±0.71	0.103
Graft type (cadaveric)	67(93.1%)	12(80.0%)	0.046
Acute rejection	9(12.5%)	5(33.3%)	0.043
**FPG (mmol/L)**			
Pretransplantation	5.55±0.81	4.55±0.62	0.040
1 year after transplantation	8.66±4.12	5.85±1.69	0.032
**TC (mmol/L)**			
Pretransplantation	5.65±1.27	3.86±1.06	0.041
1 year after transplantation	6.36±1.10	5.74±1.72	0.022
**TG (mmol/L)**			
Pretransplantation	1.81±1.13	0.94±0.32	0.002
1 year after transplantation	2.39±0.88	2.20±0.63	0.526

Abbreviations: P-NODAT: persistent-new onset diabetes after transplantation; T-NODAT: transient-new onset diabetes after transplantation; BMI: body mass index; HBV: hepatitis B virus; HCV: hepatitis C virus; CMV: cytomegalovirus; AZA: azathioprine; MMF: mycophenolate mofetil; FK506: tacrolimus; CSA: cyclosporine; FPG: fasting plasma glucose; TC: cholesterol; TG: triglyceride.

#### (3) Influence of immunosuppressant conversion on FPG level and NODAT

**Figure 2:**
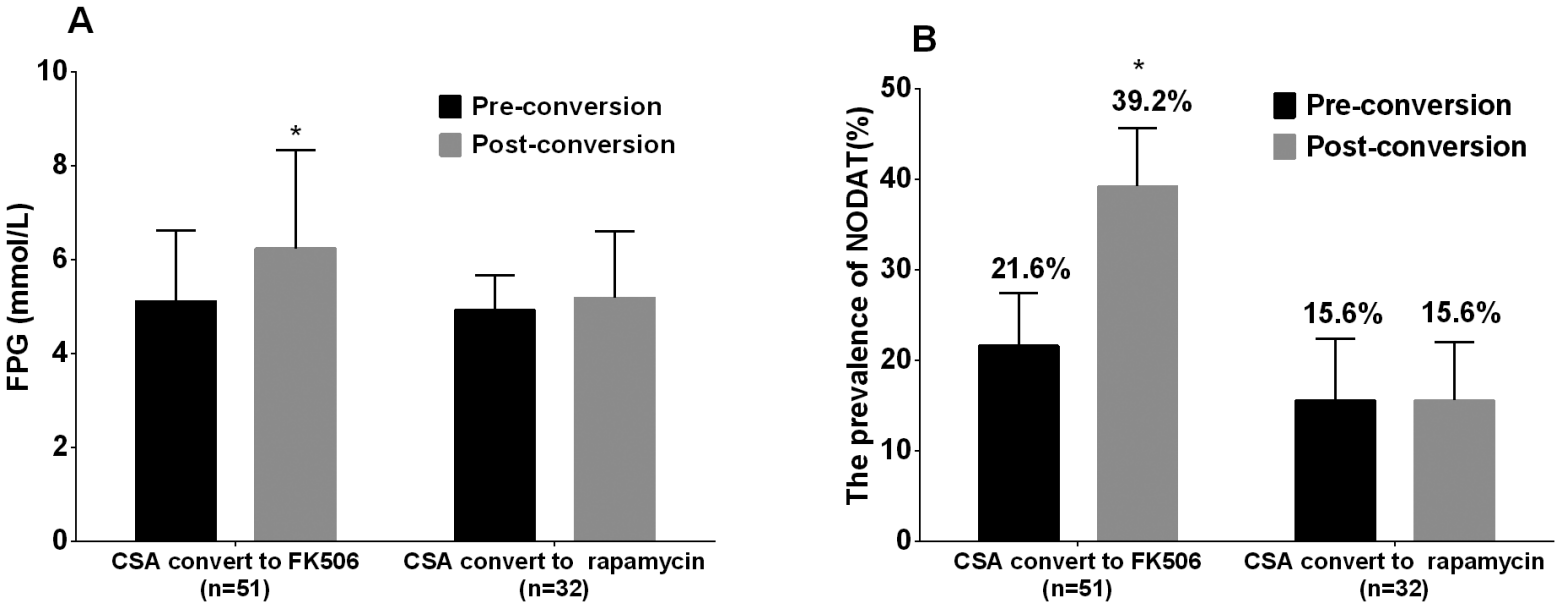
A: FPG before and after immunosuppressor conversion. B: Prevalence of NODAT before and after immunosuppressor conversion. Abbreviations: NODAT: new onset diabetes after transplantation; FPG: fasting plasma glucose; FK506: tacrolimus; CSA: cyclosporine; *P<0.05 compared with pre-conversion.

Single factor analysis indicated that adjustment of immunosuppressant therapy (conversion from CSA to FK506) is a risk factor for onset of NODAT. Hence, for the 51 patients who converted from treatment with CSA to FK506 and the 32 patients who converted from treatment with CSA to rapamycin, the FPG levels and the number of cases of NODAT before and after drug adjustment were matched separately to analyze the influence of adjustment of immunosuppressant therapy. As shown in Figures 2A and 2B, the FPG levels and the NODAT prevalence rate for the patients who converted from treatment with CSA to FK506 were significantly elevated, and both were statistically significant. However, the FPG levels and the number of cases of NODAT for patients who converted from treatment with CSA to rapamycin showed no significant changes. These results indicate that the impairment of glycometabolism was aggravated in patients who converted from treatment with CSA to FK506, but the glycometabolism of patients who converted from treatment with CSA to rapamycin was not significantly affected.

### 4. Influence of NODAT on complications and survival rate

#### (1) Influence of NODAT on complications after a renal transplant

As shown in Table 6, compared with the N-NODAT group, the NODAT group had statistically significant higher prevalence rates of postoperative infection, hypertension, and lipid metabolism disorder, whereas the prevalence rate of malignant tumors was not significantly different.

**Table 6:** Comparison of complications after transplantation between NODAT and N-NODAT.

Complications	NODAT (n = 87)	N-NODAT (n = 341)	P-value
Malignancy	5(5.7%)	11(3.2%)	0.337
Frequency of hospitalization for infection	0.77±1.227	0.48±0.754	0.036
Days of hospitalization for infection	16.67±30.25	7.42±13.23	0.006
Antihypertensive agents	1.98±1.26	1.26±1.05	<0.001
**TC (mmol/L)**			
3 months after transplantation	5.78±0.85	5.31±0.91	0.012
6 months after transplantation	5.93±1.06	5.35±1.01	0.015
1 year after transplantation	5.75±1.23	5.20±1.01	0.004
**TG (mmol/L)**			
3 months after transplantation	2.26±0.86	2.07±0.84	0.275
6 months after transplantation	2.50±1.12	1.92±0.81	0.023
1 year after transplantation	2.35±0.82	1.87±0.99	0.004

Abbreviations: NODAT: new onset diabetes after transplantation; N-NODAT: no NODAT; TC: cholesterol; TG: triglyceride.

#### (2) Influence of NODAT on the survival rate of renal transplant recipients

**Figure 3:**
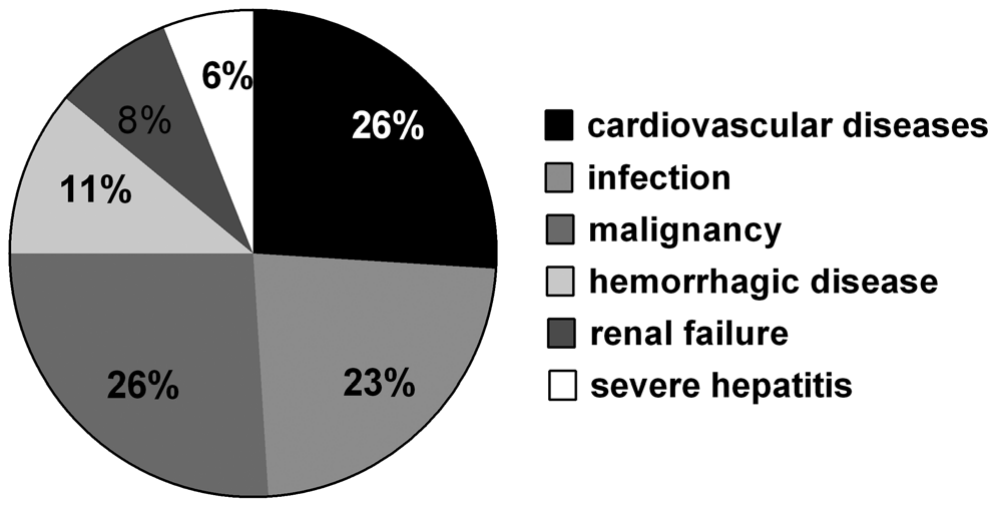
Proportion of death causes in renal transplant recipients.

As of December 2009, 34 renal graft recipients who did not have preoperative diabetes had died (7.9%); the major causes of death were cardiovascular diseases, malignant tumors, infections, bone marrow suppression, gastrointestinal bleeding ulcers and other hemorrhagic diseases, and renal insufficiency (Figure 3). Survival analysis showed that the 5-year survival rate of the NODAT group was not significantly different from that of the N-NODAT group, and the 10-year survival rate of the NODAT group was lower than that of the N-NODAT group with no statistical difference (Table 7). The previously mentioned results suggest that after an average follow-up time of 5.65 ± 3.68 years, there were no significant differences in the postoperative survival rate and survival time between the NODAT group and the N-NODAT group. Potential risk factors that might affect the death of renal transplant recipients were analyzed using Cox regression analysis (Table 8) after calibrating for age, year of transplant, postoperative onset of tumor, postoperative infection, and other risk factors that might affect the death of patients. The mortality hazard ratio of NODAT was 1.216 (95% confidence interval, 0.804–1.840), but it was not statistically significant (P  =  0.354).

**Table 7:** Comparison of risk survival rateand survival time between NODAT and N- NODAT (P = 0.959).

Group	N	5-year survival rate	10-year survival rate	Mean survival time (month)	95% confidence interval
NODAT	87	93%	81%	171.800±8.010	156.106–187.511
N-NODAT	341	93%	85%	172.160±5.130	162.090–182.232

Abbreviations: N: number;NODAT: new onset diabetes after transplantation; N-NODAT:no NODAT.

**Table 8:** Cox regression analysis of risk factors affecting death.

Parameter	HR	P-value	95% confidence interval
Age	1.040	0.017	1.007–1.075
NODAT	1.216	0.354	0.804–1.840
Malignancy	3.463	0.003	1.518–7.899
Days of hospitalizationfor infection	1.019	0.001	1.008–1.031
Cadaveric graft	1.650	0.647	0.193–14.112

Abbreviations: NODAT: new onset diabetes after transplantation.

## Discussion

### 1. Prevalence and outcome of NODAT

The prevalence rate of diabetes in patients with NODAT is significantly elevated compared with the normal population [24]. In a recent study [25] in which 209 renal transplant recipients underwent OGTT, the prevalence rates of NODAT, impaired glucose tolerance, and IFG were 19%, 14%, and 17%, respectively. The prevalence rate of NODAT in our study was 20.32%, which is close to the prevalence rate reported in the literature. In this study, 15 patients (17.2% of all cases of NODAT) regained a normal FPG level during follow-up. It has been reported [26], [27] that one-third to one-half of patients will spontaneously remit with time. However, clinical observation indicates that impaired glycometabolism cannot be reversed in all patients with NODAT [27]. Our study found that patients with P-NODAT have more recognized adjustable or nonadjustable risk factors, whereas patients with T-NODAT have a higher prevalence rate of AR compared with patients with P-NODAT (P  =  0.043), indicating that AR might be a risk factor for the onset of T-NODAT. The outcome is similar to the results reported in previous literature [28].

### 2. Risk factors for NODAT

The meta-analysis by Montori [29] showed that nonadjustable risk factors clearly related to NODAT include age, black or Hispanic ethnicity, and HCV infection. Seventy-four percent of the difference in the prevalence rate of NODAT occurring within the first year after a transplant can be explained by the type of immunosuppressant used.

#### (1) Glucocorticoids

There is evidence that treatment with glucocorticoids is the greatest risk factor for NODAT [30]. In this study, there were no differences in the daily doses at each time point within 1 year after surgery between the NODAT group and the N-NODAT group. Although postoperative withdrawal of corticosteroid therapy is still controversial in clinical studies of a renal transplant, it is commonly recognized that postoperative short-term pulsed therapy and low-dose maintenance therapy are not only safe but also reduce the risk of NODAT [31].

#### (2) Calcineurin inhibitors

CSA and FK506 are both calcineurin inhibitors (CNIs) with similar mechanisms. Studies have shown that CNI is closely correlated with glucometabolic disorders and diabetic complications in organ transplantation [32]–[35], [37]–[42]. Animal and clinical studies have demonstrated that CSA has toxic effects on pancreatic β cells [34]–[36], especially CSA formulations improved by micro-emulsion technology to achieve better oral absorbability and a higher level of drug exposure [37]. The application of CSA micro-emulsion helps explain our finding that the prevalence rate of AR significantly decreased but the prevalence rate of NODAT did not decrease correspondingly. Studies have shown that NODAT is positively correlated with CSA concentration [38], [39]. Single factor analysis in this study showed that the patient's CSA peak concentrations 6 months and 12 months after surgery are risk factors for NODAT, indicating that exposure to a high concentration of CSA in the early postoperative stages after a renal transplant is a risk factor for NODAT. Similarly, there are also animal and clinical studies which have demonstrated that FK506 has toxic effects on pancreatic β cells [36], [40], [41]. Many studies pointed out that FK506's effect on glucose metabolism was dose-dependent. With the increased use of FK506, the occurrence of NODAT was on the rise [42]. Another study [43] showed that the prevalence rate of NODAT was 20.2% higher when using FK506 instead of CSA. In our study, due to the risk of NODAT with FK506 therapy and economic concerns, our center tends to use CSA as an immunosuppressant regimen. Conversion from treatment with CSA to FK506 is a risk factor for NODAT. This also indicates that FK506 is a risk factor for NODAT, which is consistent with reports in the literature [40]–[43]. The FK506 trough concentrations in the NODAT group at each time point within 1 year after surgery showed no statistically significant difference compared with the N-NODAT group. This might be because our center followed the guidelines for the treatment and management of NODAT, closely monitored FK506 concentrations at postoperative follow-up visits, and reduced the dosage as much as possible.

#### (3) Conversion of immunosuppressant therapy

Patients had a high conversion rate of immunosuppressant regimens at our situation. In our investigation, after converting patients who were initially treated with CSA to FK506, their glycometabolism disorder was exacerbated. However, another study [44] showed that conversion in late stages from CSA to FK506 had no significant influence on glycometabolism. Patients who were converted from treatment with CSA to rapamycin in this study showed no significant changes in glycometabolism before and after conversion; however, in a study conducted by Teutonico [45], who observed the influence of CNI withdrawal with conversion to rapamycin on glycometabolism, showed that the prevalence rate of impaired glucose tolerance increased by 30%, with 4 new cases of NODAT.

#### (4) Preoperative and early-stage postoperative glycometabolism disorders

Both single factor analysis and multi-factor analysis (OR [odds ratio]  =  1.48) suggested that preoperative FPG level is clearly correlated with NODAT. International studies have also shown that preoperative glycometabolism disorder is an independent risk factor for NODAT [46]. Patients with end-stage renal disease did not show improvements in glycometabolism as renal function recovered after a renal transplant and developed NODAT. This can be explained by the “second strike” theory. Single factor analysis in our study also found that elevation of the FPG level in the first week after surgery is a risk factor for NODAT; similar results have also been reported by relevant international studies [47]. Early postoperative hyperglycemia can be used to predict postoperative glycometabolism disorder (OR  =  5.4); this is thought to be related to accumulated doses of corticosteroids after surgery.

#### (5) Others

Both single factor analysis and multi-factor analysis (OR  =  1.58) suggest that recipients of cadaveric donor kidneys are at higher risk for NODAT; this has also been reported in a related study [48]. In clinical practice, recipients of living donor kidneys are usually treated with lower doses of immunosuppressants compared with recipients of cadaveric donor kidneys, so this could be the main reason. Our investigation found that HCV and CMV are related to NODAT and that HCV infection is an independent risk factor for NODAT (OR  =  2.72), which has also been reported in related studies [49], [50]. The mechanism of diabetes caused by virus infection is unclear, but it has been speculated that insulin resistance and defects in islet cell secretion are both involved.

### 3. Influence of NODAT on patients' complications and survival rate

In our investigation, the frequency and days of hospitalization due to infections in patients with NODAT are both higher than those of patients with N-NODAT. Use of immunosuppressants after an organ transplant reduces the resistance of the body to exogenous infections, and recipients with concomitant NODAT are more vulnerable to infections. This is considered to be related to the lower chemotaxis, migration, and phagocytic function of neutrophil granulocytes in diabetic patients compared with healthy people [51]. In this study, patients in the NODAT group had more severe postoperative concomitant hypertension and lipid metabolism disorder compared with the N-NODAT group, that is, an increased prevalence rate of metabolic syndrome (MS). Another study showed that MS and NODAT were significantly correlated after a renal transplant [52]. A study by Porrini et al [53] found that patients with NODAT after a renal transplant were more likely to have MS during follow-up, with a lower human/renal survival rate. Several studies have shown the adverse effects of NODAT on renal transplantation, which is considered the second most influential factor of long-term survival after acute and chronic rejections [53]–[55]. In this study, the 5-year survival rates of the NODAT and N-NODAT groups were both 93%, and the 10-year survival rates were 81% and 85%, respectively. The difference between the 2 groups was not statistically significant (P  =  0.959). This is slightly different from the results reported in the literature [28], [56]. We consider the differences may be due to different inclusion criteria and duration of follow-up [57].

We recognize limitations of our study. First, the early preoperative OGTT data from renal transplant recipients at our hospital were not complete. The postoperative diagnosis of NODAT was only based on FPG level, so the prevalence rate of NODAT might be biased. On the one hand, diabetic patients with normal preoperative FPG but high 2-hour postprandial blood glucose levels, who met the diabetes diagnostic criteria, were not excluded, which may have caused bias and increased the prevalence rate of NODAT. On the other hand, the prevalence rate of glycometabolism disorders in our patients might have been underestimated, especially impaired glucose tolerance. Second, blood glucose, FPG, 2-hour postprandial blood glucose, and glycosylated hemoglobin levels should be routinely tested postoperatively and monitored on follow-up. Third, we believe that the homeostasis model of insulin resistance should be used to assess the insulin resistance level of patients; however, relevant data were only available from a small number of patients. Fourth, this was a single-center study and thus we are unable to reach a general conclusion. Fifth, due to the significantly extended survival time of renal transplant recipients, sample sizes should be enlarged and follow-up time should be extended as much as possible. Sixth, although the influence of NODAT on the long-term survival of patients has not emerged, its influence on the cardiovascular system might have already appeared, so extensive cardiovascular examinations should be performed to evaluate cardiovascular influences on NODAT.

In conclusion, NODAT is a severe metabolic complication after an organ transplant and has many adverse, long-term influences on the patient's life. Certain issues still need further study. First, the pathogenesis of NODAT is still unknown. Inflammation, dimethylarginines and homocysteine are recently reported to participate in the pathogenesis of diabetes, while few studies focus on pathogenesis of NODAT [51], [58], [59], [60]. Deep researches on pathogenesis of NODAT are needed. Second, more optimized immunosuppressant regimens need to be studied and developed to reduce the effects of risk factors for NODAT and thus reduce the prevalence of NODAT. At last, transplant recipients treated with long-term immunosuppressant therapy should be strictly followed up to assess the prevalence rate, type, and risk factors for long-term complications of diabetes.
